# Viral Vectors in Gene Therapy

**DOI:** 10.3390/diseases6020042

**Published:** 2018-05-21

**Authors:** Kenneth Lundstrom

**Affiliations:** PanTherapeutics, CH1095 Lutry, Switzerland; lundstromkenneth@gmail.com; Tel.: +41-79-776-6351

**Keywords:** prevention, therapy, immunotherapy, gene silencing, clinical trials, approved drugs

## Abstract

Applications of viral vectors have found an encouraging new beginning in gene therapy in recent years. Significant improvements in vector engineering, delivery, and safety have placed viral vector-based therapy at the forefront of modern medicine. Viral vectors have been employed for the treatment of various diseases such as metabolic, cardiovascular, muscular, hematologic, ophthalmologic, and infectious diseases and different types of cancer. Recent development in the area of immunotherapy has provided both preventive and therapeutic approaches. Furthermore, gene silencing generating a reversible effect has become an interesting alternative, and is well-suited for delivery by viral vectors. A number of preclinical studies have demonstrated therapeutic and prophylactic efficacy in animal models and furthermore in clinical trials. Several viral vector-based drugs have also been globally approved.

## 1. Introduction

After genuine excitement in the 1990s of the potential of gene therapy providing more or less unlimited opportunities to cure the majority of human diseases, a severe setback was encountered when adenovirus vectors were employed for the treatment of the non-life threatening disease ornithine transcarbomylase resulting in the death of a young patient [1]. Moreover, although success was achieved in the treatment of children with severe combined immunodeficiency (SCID), the integration of the retrovirus-delivered therapeutic gene into the *LMO2* proto-oncogene region triggered development of leukemia in some individuals [2,3]. These two setbacks were clearly associated with inexperience in the design of clinical trials and perhaps an overoptimistic attitude toward the safety issues related to the delivery of viral vectors for gene therapy applications. However, the outcome was severe skepticism toward gene therapy in general, which resulted in a decrease in funding, initiation of clinical trials, and support from the pharmaceutical and biotech industry. Luckily, hardcore gene therapists showed strong commitment to vector engineering to address delivery and safety issues, which has contributed to the second coming of gene therapy.

Today, both viral and non-viral vectors have seen a renaissance in innovative modifications and applications in both preclinical and clinical settings. As this review focuses on viral vectors, non-viral gene therapy approaches will not be discussed further. Instead, an overview on different viral vectors based on adenoviruses, adeno-associated viruses (AAV), alphaviruses, flaviviruses, herpes simplex viruses (HSV), measles viruses, rhabdoviruses, retroviruses, lentiviruses, Newcastle disease virus (NDV), poxviruses, and picornaviruses will be discussed. However, the large number of viral vectors evaluated for a number of disease indications only allows the presentation of gene therapy applications through selected examples. 

## 2. Viral Vectors

The spectrum of viral vectors is very broad including both delivery vehicles developed for transient short-term and permanent long-term expression. Moreover, the types of vectors are represented by both RNA and DNA viruses with either single-stranded (ss) or double-stranded (ds) genomes. The main groups of viral vectors applied for gene therapy are summarized below and in Table 1, followed by examples of both preclinical (Table 2) and clinical findings (Table 3). Finally, the approval of viral vector-based drugs is discussed.

### 2.1. Types of Vectors

The most applied viral vectors are certainly based on adenoviruses [4]. Naked dsDNA adenoviruses possess a packaging capacity of 7.5 kb of foreign DNA providing short-term episomal expression of the gene of interest in a relatively broad range of host cells. The original adenovirus vectors generated strong immune responses, whereas the so-called gutted second and third generation vectors containing deletions have proven to elicit substantially reduced immunogenicity [5]. Much attention has been paid to engineering packaging cell lines for large scale production of recombinant particles of Good Manufacturing Practice (GMP)-grade to support clinical trials [6]. AAV vectors carry a small ssRNA genome, which allows packaging of only 4 kb inserts [7]. Generally, AAV is considered to generate low pathogenicity and toxicity and provides long-term transgene expression through chromosomal integration [8]. One limitation of using AAV relates to the immune response triggered by repeated administration [9]. This problem has been addressed by applying a different AAV serotype for each re-administration. Another issue relates to the limited packaging capacity of foreign DNA into recombinant AAV particles [10]. This shortcoming has been addressed by engineering dual AAV vectors [11].

Herpes simplex viruses (HSV) are large enveloped dsDNA viruses characteristic of their lytic and latent nature of infection, which result in life-long latent infection of neurons and allows for long-term transgene expression [12]. Deletion of HSV genes has generated expression vectors with low toxicity and an excellent packaging capacity of >30 kb foreign DNA [13]. In contrast to HSV, retroviruses possess a ssRNA genome with an envelope structure [14]. Typically, retroviruses are randomly integrated into the host genome, which has been problematic, as previously described, in the therapy of SCID patients [2,3]. However, this shortcoming has triggered the development of safer vectors showing targeted integration and also improved helper cell lines [15]. Retroviruses can accommodate up to 8 kb of foreign inserts and have represented the gold standard vectors for long-term gene therapy applications. One drawback of retroviruses is their incapability to infect nondividing cells, which has enhanced the interest in application of lentivirus vectors for gene therapy. Although lentiviruses belong to the family of retroviruses, they have the capability of infecting both dividing and nondividing cells providing low cytotoxicity [16,17]. Possessing the same packaging capacity and chromosomal integration as conventional retroviruses, lentiviruses have become attractive for therapeutic applications requiring long-term expression.

Self-amplifying ssRNA viruses comprise of alphaviruses (Semliki Forest virus, Sindbis virus, Venezuelan equine encephalitis virus, and M1) and flaviviruses (Kunjin virus, West Nile virus, and Dengue virus) possessing a genome of positive polarity [18]. In contrast, rhabdoviruses (rabies and vesicular stomatitis virus) and measles viruses carry negative strand genomes [18]. Most of the self-amplifying RNA viruses possess a packaging capacity of 6–8 kb, and generate high levels of short-term transient gene expression [19]. Additionally, the ssRNA paramyxovirus Newcastle disease virus (NDV) replicates specifically in tumor cells and has therefore been frequently applied for cancer gene therapy [20]. Moreover, oncolytic cancer cell targeting vectors have been engineered for many of the listed ssRNA viruses above [21]. Another family of nonenveloped ssRNA viruses, namely Coxsackieviruses belonging to Picornaviridae, have been applied as oncolytic vectors [22,23].

Also, poxviruses and especially vaccinia viruses have been applied as delivery vectors [24]. The characteristic feature of poxviruses is their dsDNA genome, which can generously accommodate more than 30 kb of foreign DNA. Tumor-selective replication-competent poxvirus vectors have been engineered causing necrosis in nonhuman primates [25]. Additionally, vaccinia vectors, which replicate in tumor cells without damaging normal cells, were engineered by deletions in the thymidine kinase (TK) and vaccinia growth factor (VGF) genes [26].

### 2.2. Preclinical Studies

#### 2.2.1. Adenoviruses

Due to the many gene therapy applications of a number of viral vectors evaluated in preclinical animal models, only some examples can be presented here (Table 2). In this context, oncolytic adenoviruses have shown great promise in cancer therapy [27]. For instance, an oncolytic adenovirus engineered with a pancreatic cancer-targeting ligand SYENFSA (SYE), specifically infected and replicated in cancer cells, but not normal cells, provided effective oncolysis of pancreatic ductal adenocarcinoma PDAC) [28]. The AdSur-SYE vector, regulated by the survivin promoter, also showed high transduction efficiency in pancreatic neuroendocrine tumors (PNETs) [29]. Intratumoral administration of AdSur-SYE resulted in complete regression of subcutaneous tumors in a mouse model. In another approach, chimeric Adenovirus type 5 and type 3 vectors, which can selectively replicate in cancer cells, have been engineered [30]. Providing simultaneous expression of the secreted melanoma differentiation associated gene-7 (MDA-7) and interleukin-12 (L-24) from the chimeric Ad5/3 vector generated selective tumor cell death after intratumoral injection in animal models. Moreover, therapeutic activity was also confined to noninfected distant tumors due to the so-called “bystander anti-tumor activity”. To further enhance the therapeutic efficacy, the chimeric Ad5/3 vector was encapsulated in microbubbles for “stealth delivery”. Ultrasound treatment released and allowed replication of the vector, which together with secretion of MDA-7/IL-24 enhanced therapeutic activity, including promotion of apoptosis and inhibition of tumor angiogenesis. Due to the generally limited duration of therapeutic activity of adenovirus-based gene therapy, hybrid adenovirus vectors utilizing the Sleeping Beauty transposase system or clustered regularly interspaced short palindromic repeats (CRISPR) associated protein-9 nuclease have been used for chromosomal integration and permanent gene editing, respectively [31]. Oncolytic adenovirus vectors have also been used in combination with the expression of immunomodulatory proteins [32]. This approach can change the tumor microenvironment from immune-suppressive to immune-vulnerable due to activation of cytotoxic T cells. In another approach, the oncolytic adenovirus Enadenotucirev, an Ad11p and Ad3 chimeric vector, has demonstrated selective propagation and killing of tumor cells [33]. Due to the inability of replication in animal cells, Enadenotucirev was evaluated in a panel of primary human cells, which demonstrated >100-fold higher viral genome levels in tumor cells than in normal cells [33]. Furthermore, intravenous tolerability was assessed in mice. The resistance to inactivation by human blood components will potentially enable intravenous vector administration.

#### 2.2.2. Adeno-Associated Viruses

The X chromosome-linked neurodevelopmental disorder named Rett Syndrome (RTT) has been targeted by AAV vectors in a mouse model for RTT [34]. AAV vectors expressing the transcription regulator methyl CpG-binding protein 2 (MeCP2) delivered directly to the cerebrospinal fluid (CSF), showed dose-dependent side effects, but also extended survival of RTT mice. Moreover, the fatal neurodegenerative Huntington’s disease (HD) has been evaluated for AAV-based therapy in a HD mouse model [35]. Transgenic HD sheep expressing the full-length human huntingtin (HTT) gene were injected with AAV9 miRNA targeting exon 48 of the human HTT mRNA. The outcome was reduced human HTT mRNA and 50–80% HTT protein in the striatum, indicating safe and effective gene silencing. Cystic fibrosis has been targeted by AAV-based expression of the cystic fibrosis transmembrane conductance regulator (CFTR) in a number of animal models showing a good safety profile, although no clear clinical benefits [36]. Recently, the AAV1 and AAV5 serotypes were tested using a dual-luciferase reporter system based on firefly and Renilla luciferases, respectively [36]. Both AAV1 and AAV5 were delivered into lungs of Rhesus macaques by microspraying, which resulted in a 10-fold higher vector genome number of AAV1 than AAV5. However, the AAV1-based luciferase activity was not statistically higher in comparison to AAV5. Moreover, serum neutralizing antibodies showed a dramatic increase for both AAV serotypes. There were no adverse events, indicating safe administration of AAV, which supports additional clinical trials, especially with the more lung-tropic AAV1 serotype. In another approach, AAV vectors have been applied for the treatment of Duchenne and limb girdle muscular dystrophies [37]. Furthermore, dual AAV technology allowed the expression of a 7 kb canine ∆H2-R15 mini-dystrophin gene using a pair of dual AAV vectors [38]. The AAV9 was administered to the extensor carpi ulnaris muscle in a canine model for Duchenne muscular dystrophy. The outcome was widespread mini-dystrophin expression, restoration of dystrophin-associated glycoprotein complex, reduced muscle degeneration, and improved myofiber size distribution. In the context of hemophilia A, liver-specific promoter and enhancer elements with a codon-optimized human coagulation factor VIII (hFVIII) gene have been engineered [39]. One promoter-enhancer construct with high hFVIII immunogenicity was evaluated in an FVIII knockout mouse model applying AAV8, AAV9, AAVhu37, and AAVrh64R1 vectors. Based on the generation of anti-hFVIII antibodies, the vectors were divided into one group, where less than 20% of mice (AAV8 and AAV9) and the other with more than 20% of mice (AAVrh10, AAVhu37 and AAVrh64R1) generated anti-hFVIII antibodies.

#### 2.2.3. Herpes Simplex Viruses

Due to the long-term effect, HSV vectors have found many applications in various disease areas. For instance, HSV-based expression of proinflammatory cytokines has proven useful in treatment of painful diabetic neuropathy [40]. In this context, continuous delivery of HSV-IL-10 into the nerve fibers of mice with type I diabetes blocked nociceptive and stress responses in transduction of the dorsal root ganglion (DRG) [40]. It was suggested that macrophage activation in the peripheral nervous system is involved in the pathogenesis of pain and that HSV-based cytokine expression inhibited the development of painful neuropathy. In another approach, administration of nonreplicating HSV vectors expressing growth factors in the skin of mice resulted in the transduction of DRGs and prevented the progression of sensory neuropathy without causing any side effects [41]. Related to cancer, oncolytic HSV vectors have been applied in several preclinical studies [42]. The genome of the HSV-1 HF10 vector includes nonengineered deletions and mutations and frame-shift mutations lacking the expression of UL43, UL48.5, UL55, UL56, and latency-associated transcripts, while demonstrating overexpression of UL53 and UL54. HSV-1 HF10 replicates efficiently in tumor cells causing extensive cytotoxic damage. Moreover, activated CD4^+^ and CD8^+^ T cells and natural tumor killer cells were induced in tumors resulting in significant tumor growth reduction and prolonged survival. Oncolytic HSV-2 vectors have also been evaluated in animal studies on colon cancer cells and cancer stem-like cells (CSLCs) and are known to be tumorigenic and responsible for cancer recurrence and metastasis [43]. Significant inhibition of tumor growth was observed after administration of oncolytic HSV-2 vectors.

#### 2.2.4. Retroviruses

Retroviruses present the classic approach for long-term gene therapy applications and the first human gene therapy trial involved implantation of autologous bone marrow cells transduced ex vivo with gamma retrovirus vectors [44]. More recently, attention has been paid to target dendritic cells (DCs) by engineering of vectors with DC-specific promoters or by retargeting vector tropism [45]. Also, transduction of hematopoietic stem cells has supported antigen-specific immune tolerance. In another immunotherapy approach, the low gene transduction efficiency of 50% of chimeric antigen receptor-expressing T (CAR-T) cells was improved to more than 90% by optimization of precultivation conditions and antibody stimulation [46]. The transduced CAR-T cells showed antigen-specific cytotoxic activity and secreted cytokines by antigen stimulation. Related to cancer therapy, the nonlytic amphotropid retroviral replicating vector (RRV) Toca 511 encoding yeast cytosine deaminase (CD) was delivered to tumors in orthotopic glioma models [47]. When combined with 5-fluorocytosine (5-FC), CD in infected tumor cells converts 5-FC to 5-fluorouracil (5-FU) leading to cell death. Intravenous or intracranial administration of Toca 511 provided long-term survival in immune-competent mice after combination treatment with 5-FC. Prolonged survival was also observed in animals with pre-existing immune response to the vector, which supports the potential of readministration. The self-inactivating gammaretroviral vector (SINfes.gp91s), containing the codon-optimized transgene (gp91(phax)) and the promoter for the X-linked form of the immunodeficiency named chronic granulomatous disease (CGD), was demonstrated to protect X-CGD mice from challenges with *Aspergillus fumigatus* [48].

#### 2.2.5. Lentiviruses

In the case of lentivirus-based gene therapy, a lentiviral vector carrying the human pyruvate kinase deficiency (hPKD) promoter and the PKLR gene was employed for addressing the monogenic metabolic disease PKD caused by mutations in the pyruvate kinase isoenzymes L/R (PKLR) gene [49]. When mouse hematopoietic stem cells (HSCs) transduced with lentivirus were transplanted into myeoblated PKD mice, the erythroid compartment was normalized providing a corrected hematological phenotype and reversion of organ pathology. Furthermore, analysis of the genomic insertion sites for the lentivirus vector in transplanted hematopoietic cells indicated no presence of genotoxicity. Lentivirus vectors have also been subjected to gene therapy applications of RNA silencing in the CNS [50]. Related to Parkinson’s disease (PD), the misregulation and overexpression of α-synuclein leading to its accumulation in neurons was counteracted by lentivirus-based RNA interference (RNAi) in the human dopaminergic cell line SH-SY5Y and in neurons in rat striatum [51]. Moreover, in another approach, the PD-related transcriptional upregulation of the GABA-producing enzyme glutamate decaorboxylase 1 (GAD1) or GAD67 was successfully knocked down by lentivirus-mediated shRNA-miR expression in a rat model for PD, demonstrating normalized neuronal activity [52]. In the context of Alzheimer’s disease, lentivirus vectors have been applied for RNA silencing to knock down BACE1 attenuated amyloid precursor protein (APP) cleavage and β-amyloid production, resulting in reduced neurodegeneration and behavioral deficits in an Alzheimer’s disease mouse model [53]. In another approach, lentivirus-based siRNA expression showed reduced tau phosphorylation and number of neurofibrillary tangles in an Alzheimer’s disease mouse model [54]. Furthermore, lentivirus vector-based delivery of shRNAs targeting the HIV-1 coreceptor CCR5 and the R-region of the HIV-1 long terminal repeat (LTR) has been evaluated in humanized bone marrow/thymus (hu-BLT) mice [55]. The outcome was efficient inhibition of HIV infection and might provide a potential therapy against HIV. In another approach, the Cal-1 anti-HIV lentiviral vector was evaluated in pigtailed macaques [56]. Cal-1 lentivirus demonstrated safe integration and preclinical safety.

#### 2.2.6. Alphaviruses

Alphaviruses have been mainly applied in preclinical gene therapy studies for cancer treatment [57]. The particular feature is that alphavirus vectors can be delivered in the form of naked RNA, layered plasmid DNA vectors and recombinant replication-deficient or -proficient particles. In this context, local administration of a replication-proficient Semliki Forest virus (SFV) vector expressing EGFP, generated prolonged survival in mice with implanted A549 lung carcinoma xenografts [58]. In another study, SFV-IL-12-based therapy was evaluated in a syngeneic RG2 rat glioma model, which resulted in 87% reduction in tumor volume and significant extension of survival [59]. In attempts to target tumor cell replication, six micro-RNAs (miRNAs) were introduced into the SFV genome. Intraperitoneal administration of engineered SFV4-miRT124 particles in BALB/c mice resulted in glioma targeting, limited spread in the CNS and significantly prolonged survival rates [60]. Moreover, the naturally occurring oncolytic alphavirus M1 was demonstrated to selectively kill zinc-finger antiviral protein (ZAP)-deficient cancer cells and also showed high tumor tropism and potent oncolytic activity in a liver tumor model [61].

#### 2.2.7. Flaviviruses

In the context of flaviviruses, the granulocyte macrophage colony-stimulating factor (GM-CSF) expressed from a Kunjin virus vector was subjected to intratumoral administration in mice with subcutaneous CT26 colon carcinoma [62]. The treatment resulted in a cure of more than 50% of injected mice; tumors were undetectable 18 days after Kunjin-GM-CSF administration. Likewise, treatment of B16-OVA melanoma tumors led to significant tumor regression after 5 days and the cure rate in mice reached 67% [62]. Moreover, subcutaneous injection of Kunjin-GM-CSF resulted in regression of CT26 lung metastasis in BALB/c mice.

#### 2.2.8. Rhabdoviruses

Among rhabdoviruses, recombinant vesicular stomatitis virus (VSV) has been applied for preclinical gene therapy studies. The low seroprevalence in humans and robust heterologous expression profile have supported a number of vaccine approaches against human pathogens [63]. For instance, VSV vectors expressing HIV-1 Gag and Env elicited robust HIV-1 specific cellular and humoral immune responses in nonhuman primates [63]. Furthermore, vaccinated animals were protected against challenges with a pathogenic SIV/HIV recombinant. However, the neurovirulence of VSV vectors has remained an issue of concern leading to strategies of developing attenuated vectors [60]. In another approach, a chimeric VSV vector, where the VSV G envelope was replaced by a lymphocytic choriomeningitis virus glycoprotein (LCMV-GP), the chimeric vector presented no harm to normal brain cells, but efficiently eliminated brain tumor cells in several tumor models in vivo [64]. Moreover, safe systemic administration was confirmed in mice and no humoral activity against VSV was detected, which provided the basis for repeated systemic injections. In preparation for future clinical trials, the oncolytic VSV-IFNβ-NIS vector expressing interferon-β (IFNβ) and sodium iodide transporter (NIS) was evaluated in preclinical rodent models [65]. For instance, dose-dependent tumor regression was demonstrated in C57BL1/KaLwRij mice implanted with syngeneic 5TGM1 plasmacytoma tumors. However, KAS6/1 xenografts regressed at all VSV doses tested in SCID mice. Moreover, purpose-bred dogs with naturally occurring tumors were subjected to a dose-escalation study with VSV-IFNβ-NIS [66]. The intravenous maximum tolerated dose (MTD) was determined to 10^10^ TCID_50_ with mild to moderate adverse events. The VSV genome disappeared rapidly and anti-VSV antibodies were detected 5 days after administration in the blood. However, no infectious virus was detected in the plasma, urine or buccal swabs. In another study, VSV-based expression of human mucin 1 (MUC1) provided significant reduction of tumor growth in mice with established pancreatic ductal adenocarcinoma xenografts [67]. Furthermore, combination of VSV-MUC1 and gemcitabine resulted in superior therapeutic efficacy.

#### 2.2.9. Measles Viruses

Measles viruses have found a number of gene therapy applications, which have been evaluated in preclinical animal models. In this context, the oncolytic MV-Edm was engineered to express NIS, which is depleted in aggressive and radioiodine resistant anaplastic thyroid cancer (ATC) [68]. Treatment with MV-NIS confirmed NIS expression and enhanced tumor killing. In another approach, measles virus was engineered to express a yeast-based bifunctional suicide gene encoding cytosine deaminase and uracil phosphoribosyltransfrerase named super-cytosine deaminase (SCD) [69]. The chimeric protein is capable of converting the nontoxic prodrug 5-fluorocytosine (5-FC) into highly cytotoxic 5-fluorouracil (5-FU). Furthermore, 5-FU is directly converted into 5-fluorouridine monophosphate (5-FUMP), which addresses the issue of chemoresistance to 5-FU in cancer treatment. Transduction with MV-SCD showed replication and efficient lysis of human ovarian cancer cell lines and primary tumor cells. Moreover, precision-cut tumor slices from human ovarian cancer patients demonstrated efficient infection by MV-SCD. The MV-SCD also showed strong oncolytic activity in a mouse xenograft model of human hepatocellular carcinoma (HCC) [70]. Furthermore, MV-SCD generated long-term virus replication in tumor tissue and induced apoptosis-like cell death independent of intact apoptosis pathways. In another study, MV-SCD was administered intratumorally in combination with systemic 5-FU in a TFK-1 xenograft mouse model, which resulted in significant tumor reduction [71]. Moreover, tumor reduction and significant survival benefits were observed in a HuCCT1 xenograft model [71].

#### 2.2.10. Newcastle Disease Viruses

Newcastle disease virus (NDV) vectors have been frequently used in preclinical cancer therapy studies due to their oncolytic activity [72]. Although NDV vectors expressing IL-2 showed promise, comparative studies with the less toxic IL-15 have been conducted. Intratumoral injection of NDV-IL15 and NDV-IL2 in melanoma-bearing mice showed efficient suppression of tumor growth [72]. However, the 120 day survival rate was 12.5% higher after NDV-IL15 treatment than that of NDV-IL2. Likewise, the survival rate was 26.6% higher for NDV-IL15 treatment in a tumor rechallenge experiment. In another study, reverse genetics were employed on the oncolytic NDV D90 strain to generate recombinant NDVs carrying the GFP gene [73]. The rescued virus showed tumor-selective replication and induced apoptosis in tumor cells in athymic mice with implanted lung tumors. It has also been demonstrated that expression of IL-2 and tumor necrosis factor-related apoptosis inducing ligand (TRAIL) enhanced inherent antineoplasticity by inducing apoptosis [74]. The NDV-TRAIL and the bifunctional NDV-IL2-TRAIL showed superior apoptotic function in comparison to NDV-IL2. Moreover, CD4^+^ and CD8^+^ proliferation was induced and expression of TFN-α and IFN-γ antitumor cytokine expression was elicited. The NDV-IL2-TRAIL also exhibited prolonged survival in mice implanted with HCC and melanoma xenografts. In another study, the NDV Anhinga strain was applied for the expression of soluble TRAIL (NDV/Anh-TRAIL), which resulted in efficient suppression of HCC without significant cytotoxicity [75].

#### 2.2.11. Coxsackieviruses

Coxsackieviruses have been used for several gene therapy applications [23]. For instance, the coxsackievirus B3 (CVB3) expressing the human fibroblast growth factor 2 (FGF2) was injected into ischemic hindlimbs of mice showing protection from ischemic necrosis [76]. The treatment improved the blood flow in ischemic limbs for more than 3 weeks. Moreover, the recombinant CVB3 showed a drastic decrease in virulence compared to wild type CVB3. Related to cancer, Coxsackievirus A21 (CAV21) expressing intercellular adhesion molecule-1 (ICAM-1) and decay-accelerating factor (DAF) reduced tumor burden in nonobese SCID mice implanted with melanoma xenografts [77]. A single administration of CAV21 was sufficient to provide efficient oncolysis and the systemic spread of CAV21 showed efficient regression in tumors distantly located from the site of viral injection. Furthermore, the same CAV21 vector was evaluated in SCID mice implanted with T47D and MDA-MB-231-luc breast tumor xenografts [78]. A single intravenous injection generated significant regression of pre-established tumors and also targeting and elimination of metastases. Furthermore, intravenous injection of CVA21 expressing ICAM-1 and DAF in combination with intraperitoneal injection of doxorubicin hydrochloride provided significantly enhanced tumor regression in comparison to either virus or drug alone in mice with implanted MDA-MB-231 tumors [79]. Related to prostate cancer, the low pathogenic enteroviruses, CVA21, CVA21-DAFv, and Echovirus 1 (EV1), were tested in SCID mice [80]. Systemic delivery induced regression of tumor xenografts and a therapeutic dose-response was obtained for escalating doses of EV1 in the LNCaP mouse model.

#### 2.2.12. Poxviruses

Finally, poxviruses have found several applications as gene therapy vectors. For instance, vaccinia virus vectors have demonstrated potential for treatment of pancreatic cancer [81]. In this context, the PANVAC system comprising of recombinant vaccinia and fowlpox viruses, carrying the tumor-associated antigens epithelial MUC-1 and carcinomebryonic antigen (CEA) as well as T cell stimulatory molecules, have been applied [82]. Sequential subcutaneous administration of the vectors has provided induced CEA and MUC-1 CTL responses in preclinical animal models. In the case of HCC, the light-emitting recombinant GLV-2b372 vaccinia virus was injected into HCC xenografts in the flank of athymic nude mice for assessment of tumor growth and inhibition of viral biodistribution [83]. It was demonstrated that flank tumor volumes decreased by 50% 25 days after injection, while tumor volumes increased by 400% in control mice. Related to prostate cancer, NIS expression from the GLV-1h153 vaccinia virus in combination with radiotherapy was evaluated in CD1 nude mice implanted with PC3 xenografts [84]. Combination therapy was superior to individual treatments both in xenograft and immunocompetent transgenic adenocarcinoma of the mouse prostate (TRAMP) mouse models, demonstrating restricted tumor growth and improved survival rates. A vaccinia virus was engineered by mutating the F4L gene, the viral homologue of the cell-cycle-regulated small subunit of ribonucleotide reductase 2 (RRM2), which provided tumor-selective replication and cell killing [85]. It was confirmed that the F4L-mutated vector selectively replicated in immune-competent rat AY-27 and xenografted human RT122-luc orthotopic bladder cancer models, resulting in substantial tumor regression or complete ablation without causing any cytotoxicity. Moreover, antitumor immunity was established in rats cured of AY-27 tumors. Recently, a novel cowpox virus (CPXV) vector was engineered with a deletion of the thymidine kinase (TK) gene and insertion of the suicide gene FCU1, which is responsible for conversion of 5-FC into 5-FU and 5-FUMP [86]. Systemic administration of the modified CPXV vector showed accumulation in tumor cells and low infection and toxicity of normal cells. Moreover, intratumoral administration in U-87-MG glioblastoma and LoVo colon cancer models, induced relevant tumor growth inhibition. 

### 2.3. Clinical Trials

A substantial number of clinical trials have been conducted or are currently in progress applying viral vectors (Table 3). For instance, the tumor-selective chimeric Enadenotucirev adenovirus vector was subjected to intravenous delivery in 17 patients with resectable colorectal cancer, non-small-cell lung cancer, urothelial cancer and renal cancer [87]. Tumor-specific delivery was observed in most tumor samples with no treatment-related serious adverse events.

Related to hemophilia, gene therapy has been employed already for three decades, mainly focusing on AAV-based vectors [88]. In addition to discovery of pre-existing neutralizing antibodies in animal models, clinical trials have revealed that liver transaminase levels are elevated and immune-related loss of transgene expression. The mechanism of the decrease in expression levels is not fully understood, but the use of different serotypes for consecutive administration of AAV has provided improved transgene expression [9], which has resulted in long-term expression of factors VIII (FVIII) and IX (FIX) and furthermore allows a cure of severe bleedings and joint damage associated with hemophilia. In this context, 11 hemophilia gene therapy clinical trials have been conducted and six ongoing phase I/II clinical trials have applied liver-directed AAV expressing either FVIII or FIX with some success [89]. Furthermore, stem cell-based lentiviral vector delivery has proven successful in establishing sustained high level FIX expression after differentiation of adipogenic, chondrogenic, and osteoblastic cells [90], which potentially can be applied for treatment of hemophilia B. Likewise, stem cell-based lentiviral gene therapy can provide life-long production of FVIII and the potential cure of hemophilia A [89].

The oncolytic HSV HF10 vector has been subjected to clinical trials in recurrent breast cancer, head and neck cancer, unresectable pancreatic cancer, refractory superficial cancer, and melanoma [42]. The studies demonstrated high safety and a low frequency of adverse effects in treated patients. Moreover, HF10 antigens were detected 300 days after immunization in pancreatic cancer patients. Combination therapy with HF10 and ipilimumab (anti-CTLA-4) showed a good safety profile and good antitumor efficacy in a phase II trial [42]. Related to retroviruses, a clinical trial in patients with recurrent high-grade glioma (HGG) is currently in progress with the Toca 511 retrovirus [47]. Moreover, Toca 511 was subjected to an open-label, ascending dose, multicenter phase I trial in patients with recurrent or progressive HGG [91]. The overall survival was 13.6 months and statistically better, relative to an external control group. Moreover, tumor samples from patients surviving more than a year demonstrated survival-related RNA expression in correlation with treatment-related survival. Currently, a phase II/III trial with Toca 511 is in progress [92]. In another approach, a gammaretroviral vector was employed for the treatment of chronic granulomatous disease (CGD), which relates to primary immunodeficiency, resulting in an impaired antimicrobial activity in phagocytic cells [48]. The phase I/II trial revealed that although bacterial and fungal infections were transiently resolved, clonal dominance and malignant transformations compromised the therapeutic effect, suggesting that alternative vectors should be considered for delivery [48]. In another cancer-related approach MV-NIS has been approved by the FDA for human clinical trials in myeloma patients, which could provide a potential strategy for targeting iodine-resistant ATC [66]. Oncolytic vaccinia viruses have also been subjected in a phase I clinical trial in 11 patients with refractory advanced colorectal or other solid cancers [93]. The study showed neither dose-related toxicity nor any treatment-related severe adverse events. However, a strong induction of inflammatory and Th1 cytokines indicated a potent mediation of potential immunity against cancer, which supports further trials with intravenously administered vaccinia virus in combination with expression of therapeutic genes, immune checkpoint blockade, or complement inhibitors. In another study applying poxviruses, the PANVAC-VF vaccine regimen composed of a priming dose of recombinant vaccinia virus and booster doses of recombinant fowlpox virus expressing CEA, MUC-1, and a triad of costimulatory molecules (TRICOM) was subjected to subcutaneous administration in patients with advanced pancreatic cancer [94]. The safety and ability of PANVAC-VF to induce antigen-specific T cells was demonstrated [80]. However, a phase III trial targeting patients with metastatic pancreatic cancer failed to meet the therapeutic targets and was terminated [95]. In another approach, a phase I trial for direct intratumoral injection of PANVAC-VF has generated some encouraging results [96]. 

In the context of HSV-based clinical trials, the oncolytic HSV M032 vector expressing IL-12 has been subjected to a phase I dose-escalating study in patients with recurrent or progressive malignant glioma [97]. Moreover, the HSV strain G207 lacking genes essential for replication in normal cells were evaluated in patients with recurrent glioblastoma multiforme [98]. After two doses of HSV G207 (totaling 1.15 × 10^9^ pfu) no patients developed HSV encephalitis, but significant antitumor activity was observed. Furthermore, the study demonstrated safe multiple dose delivery including direct injections into the brain. In a phase I study in patients with recurrent/progressive HGG six of nine patients had stable disease of partial response and the median survival time was 7.5 months after a single-dose oncolytic HSV injection, indicating the potential for clinical response [99]. Furthermore, preclinical studies with HSV G207 have generated highly sensitive tumor killing, which support the initiation of the first-in-children study of intratumoral administration in children with recurrent or progressive supratentorial malignant tumors [100]. Alphaviruses have so far been subjected to only a limited amount of clinical trials. In this context, recombinant VEE replicon particles expressing the prostate specific membrane antigen (PSMA) were administered to patients with castration resistant metastatic prostate cancer in a phase I dose-escalation study [101]. The immunization showed no toxicity, but no PSMA-specific cellular immune response was detected with only weak signals detected by ELISA with a dose of 9 × 10^6^ IU.

Similar results occurred when immunizations were performed with 3.6 × 10^7^ IU. Despite the lack of clinical benefit and robust immune responses, immunizations elicited neutralizing antibodies, which encourages further dose optimization studies. In another approach, liposome-enveloped SFV vectors expressing IL-12 were subjected to systemic administration in a phase I study in melanoma and kidney carcinoma patients [102]. Intravenous injections provided a transient 5-fold increase of IL-12 in the plasma. Due to the encapsulation procedure, tumor targeting and protection against recognition by the host immune system was obtained, which also allowed repeated vector administration. 

NDV has been used in a number of clinical trials [103]. For instance, NDV expressing multiple tumor-associated antigens (TAAs) has been demonstrated to provide long-term survival in phase II trials in patients with ovarian, stomach, and pancreatic cancer [104]. Furthermore, melanoma patients were immunized with NDV in a randomized double-blind phase II/III trial [105]. However, the study results suggested that there were no remarkable differences between the vaccinated individuals and those in the placebo group. In a phase II study 79 patients with solid tumors were subjected to intravenous administration of the NDV PV101 strain [106]. A lower dose of 12 × 10^9^ pfu/m^2^ and an MTD of 12 × 10^10^ pfu/mL were applied, which resulted in objective response to the higher dose and progression-free survival ranging from 4 to 31 months. In another phase III trial, 335 patients with colorectal cancer were subjected to NDV immunotherapy [107]. It was demonstrated that vaccination with NDV provided prolonged survival and short-term improved quality of life.

Approaches on HIV gene therapy lentivirus vectors have been employed for targeting CCR5 by shRNA delivery [108]. The shRNAs were demonstrated to effectively inhibit CCR5 expression providing protection against HIV-1 infection in cell cultures [109]. Moreover, a self-activating lentiviral vector has been engineered to express a combination of the sh5 anti-HIV gene and the C46 antiviral fusion inhibitor peptide, which provided a synergistic effect on HIV-1 inhibition [108]. The promising results of preclinical studies triggered the first phase I clinical trial applying RNA interference to down-regulate CCR5 expression in HIV therapy [110].

Related to Coxsackieviruses, a phase I/II trial in melanoma patients with the CVA21 showed good tolerance, viral replication in tumors and increased antitumor activity [111]. The latter could be further enhanced by combination therapy with immune checkpoint blockade. In another phase II trial, CVA21 demonstrated induced immune cell infiltration in the tumor microenvironment of patients with melanoma [112]. Moreover, combination therapy of CVA21 and systemic pembrolizumab in a phase 1b study in melanoma patients showed a best overall response rate of 60% and stable disease in 27% of the patients [113]. Neither dose-limiting toxicity nor grade 3 or higher treatment-related adverse events were observed.

In the context of cystic fibrosis, a pseudotyped lentivirus vector with a fusion protein (F)/hemagglutinin-neuraminidase (HN) was optimized for promoter/enhancer sequences and evaluated in mice, and human air–liquid interface (ALI) cultures in preparation for a first-in-man CF clinical trial [114]. The lentivirus vector carrying a hybrid cytosine guanine dinucleotide (CpG)-free CMV enhancer/elongation factor 1 alpha promoter (hCEF) expressed functional CFTR, retained 90–100% transduction efficiency in clinically relevant delivery devices and showed acceptable toxicity and integration site profiles to support the initiation of a clinical trial in CF patients.

### 2.4. Approved Drugs

The first viral-based gene therapy drugs were approved some time ago in China [115]. In this context, oncolytic adenoviruses expressing the *p53* gene (Gendicine^TM^) [115] and AdH101 containing the E1b-55K deletion [116] are used for treatment of cancers with mutated p53 and head and neck cancer, respectively. Gendicine^TM^ has been used for 12 years in more than 30,000 patients with an exemplary safety record and has provided significantly better responses compared to standard therapies when combined with chemotherapy and radiotherapy [117]. Moreover, the progression-free survival times were significantly extended.

Furthermore, a second-generation oncolytic HSV vector expressing GM-CSF has been approved in the US and Europe for melanoma treatment [118,119]. Unfortunately, although the AAV-based Glybera drug was approved for treatment of the rare inherited disorder lipoprotein lipase deficiency, the high costs and limited demand forced the withdrawal from the market [120].

Additionally, several other viral-based drugs will most likely be on the market in the near future. For instance, oncolytic VV JX-594 (pexastimogene devacirepvec) for hepatocellular carcinoma treatment [121], Ad CG0070 expressing GM-CSF for bladder cancer [122], and the wild type retrovirus-based pelareorep (Reolysin^®^) [123] for head and neck cancer are at late-stage development. Moreover, the third generation oncolytic HSV-1 G47∆, which was subjected to a phase II glioblastoma study [124], has been further designated as a “Sakigake” breakthrough therapy, which will provide priority reviewing and fast-track approval [118]. 

## 3. Conclusions

In summary, the field of gene therapy has seen some significant progress with nearly 3000 clinical trials conducted by 2017 [125]. Not surprisingly, 64.6% of the trials relate to cancer therapy. Furthermore, 10.5% focus on monogenic diseases, 7.4% on infectious diseases, and 7.4% on cardiovascular diseases. Interestingly, nearly 70% of the trials have utilized viral vectors. Although recent developments in gene manipulation methods, such as CRISPR, and more efficient delivery methods for nonviral vectors, viral vectors still remain attractive.

In attempts to further improve the applicability of viral vectors, a number of modifications have been introduced. For instance, the issue of insertional oncogenesis of retroviruses leading to activation of *LMO2* or other oncogenes has been a major concern in treatment of SCID [2,3]. For this reason, a new assay has been developed for assessing vector safety related to insertions into the *LMO2* locus and other T-cell proto-oncogenes [126]. It was revealed that gamma-retrovirus vectors with full viral long-terminal repeats were most prone to *LMO2* pathway activation. On the other hand, lentiviral vectors showed a significantly lower tendency of proto-oncogene activation. The third generation lentiviruses have also contributed to improved delivery and safety [127]. For instance, deletion of the viral *tat* gene makes the vector replication-incompetent [128]. Furthermore, lentiviral vector packaging functions have been placed on three plasmids instead of two to reduce the risk of recombination. Modifications of the 3′ LTR prevents integrated genes from being repackaged providing a self-inactivating system. Additionally, VSV-G pseudotyped lentiviral vectors allow transduction of a much wider range of cell type and the enhanced vector stability, which facilitates high titer vector concentration by ultracentrifugation. Another issue relates to host genetic variations affecting transgene expression from lentivirus vectors, which was studied in 12 collaborative cross mouse strains [129]. Total body and hepatic luciferase expression was monitored in female mice after administration of a lentivirus vector with a liver-specific promoter. The study revealed major strain-specific transduction, vector biodistribution, and maximum luciferase expression and kinetics, highlighting the importance of genetic variation and the need for redesigning preclinical studies.

The future of viral-based gene therapy, even taking a cautious approach, sounds bright. The approval of several drugs for the treatment of different conditions by applying various viral vectors provides substantial flexibility and alternative strategy options. The progress made in vector engineering and safety development has also put viral vectors in a favorable position. However, both preclinical and clinical studies have confirmed that there is no single universal viral vector suitable for the treatment of all indications. The choice of vector is therefore influenced by factors such as level of expression of the therapeutic agent, duration of expression, and even personal experience and preference of the researchers/clinicians. As personalized medicines have become an essential part of modern drug development, viral vectors should also find potential opportunities in this area. In this context, improved adenovirus vectors could be engineered for personalized drug delivery [130]. In another approach, a library of tumor antigen-specific T-cell receptor (TCR) genes from frozen tumor biopsies were introduced into a retroviral vector, which allowed rapid generation of therapeutic personalized antitumor T-cell products [131].

It is therefore not useful to try to make suggestions of which viral vector system to use, but rather encourage parallel development of several systems and then make appropriate decisions based on publications and previous experience. Anyway, viral-based gene therapy has developed substantially and is on the way to becoming a key treatment in modern medicine. 

## Figures and Tables

**Table 1:** Examples of viral vectors applied for gene therapy.

Virus	Genome	Insert Capacity	Features	Reference
**Adenoviruses**	dsDNA	<7.5 kb	broad host range	[4–6]
Ad5			transient expression	
			strong immunogenicity	
**AAV**	ssDNA	<4 kb	relatively broad host range	[7–10]
AAV2, 3, 5, 6, 8, 9			slow expression onset	
			chromosomal integration	
			immune response	
**Herpes simplex**	dsDNA	>30 kb	broad host range	[12,13]
HSV1, HSV			latent infection, long-term expression	
			low toxicity, large insert capacity	
**Retroviruses**	ssRNA	8 kb	transduces only dividing cells	[14,15]
MMSV			long-term expression	
MSCV			random integration	
**Lentiviruses**	ssRNA	8 kb	broad host range	[16,17]
HIV-1, HIV-2			low cytotoxicity, integration	
			long-term expression	
**Alphaviruses**	ssRNA	8 kb	broad host range	[54]
SFV, SIN,			extreme transient expression	
VEE, M1			low immunogenicity	
			neuron- and glial-specific mutants	
**Flaviviruses**		6 kb	relatively broad host range	
Kunjin, West Nile,	ssRNA		transient expression	[18]
Dengue virus			packaging system	
**Rhabdoviruses**	ssRNA	6 kb	relatively broad host range	[18]
Rabies, VSV			high transient expression	
			low immunogenicity	
**Measles virus**	ssRNA	6 kb	transient expression	[18]
MV-Edm			oncolytic strains	
**Newcastle disease**	ssRNA	6 kb	replication in tumor cells	[20,21]
**Virus**			improved oncolytic vectors	
**Poxviruses**	dsDNA	>30 kb	broad host range, large inserts	[24–26]
VV			replication-competent vectors	
**Picornaviruses**	ssRNA	6 kb	oncolytic strains	[22,23]
Coxsackievirus				

AAV, adeno-associated virus; HIV, human immunodeficiency virus; HSV, herpes simplex virus; MMSV, Moloney murine sarcoma virus; MSCV, murine stem cell virus; SFV, Semliki Forest virus; SIN, Sindbis virus; VEE, Venezuelan equine encephalitis virus; VSV, vesicular stomatitis virus; VV, vaccinia virus.

**Table 2:** Examples of gene therapy applications in animal models.

Virus Vector	Disease	Target	Response	Reference
**Adenovirus**				
Ad-SYE	pancreatic CA	SYE	targets pancreatic tumors	[28]
AdSur-SYE	neuroendocrine CA	SYE-survivin	targets PNETs providing complete regression	[29]
Ad5/3-MDA7/IL-24	breast CA	MDA7/IL-24	selective tumor death, bystander effect, microbubble encapsulation	[30]
Ad-SB	cancer	Hybrid Ad	chromosomal integration	[31]
Ad-CRISPR	glioma	CRSIPR	T cell activation	[32]
oncolytic Ad	glioma	+ immunomodulatory proteins	modified tumor microenvironment	[32]
**AAV**				
AAV-MeCP2	RTT	MeCP2	dose-related toxicity, extended survival of mice	[34]
AAV1, AAV5	HD	HTT miRNA	gene silencing of HTT in transgenics	[35]
Dual AAV9	CT	luciferase	reporter expression in macaque lung	[36]
	DMD	mini-dystrophin	mini-dystrophin expression,	[38]
AAV8, AAV9,	hemophilia	FVIII	reduced muscle degeneration	
AAVrh10, AAVhu37			anti-FVIII antibodies	[39]
**HSV**				
HSV1, HSV2	PDN	IL-10	blocked nociceptive and stress responses	[40]
	SN	growth factors	prevention of SN	[41]
HSV-1 HF10	cancer	HF10	tumor regression, improved survival	[42]
Oncolytic HSV-2		HSV-2	tumor growth inhibition	[43]
**Retroviruses**				
RRV/ Toca 511	glioma	CD	combination therapy with 5-FC prolonged survival in mice	[47]
GRV	X-CGD	SINfes.gp91s	protection against *A. fumigatus*	[48]
**Lentiviruses**				
HIV-1, HIV-2	PKD	PKLR	corrected hematological phenotype	[49]
	PD	RNAi	down-regulated α-synuclein	[51]
		GAD67	normalized neuronal activity	[52]
	AD	RNAi	reduced neurodegeneration	[53]
		siRNA	reduced tau phosphorylation	[54]
	HIV	shRNA	inhibition of HIV infection	[55]
	SHIV	Cal-1	safe integration	[56]
**Alphaviruses**				
SFV	lung CA	EGFP	prolonged survival	[58]
	glioma	IL-12	tumor regression,	[59]
		miRNAs	prolonged survival	[60]
M1	liver CA	onolytic M1	tumor targeting, prolonged survival tumor growth inhibition	[61]
**Flaviviruses**				
Kunjin virus	colon CA	GM-CSF	tumor regression	[62]
**Rhabdoviruses**				
VSV	AIDS	HIV-1 Gag/Env	protection against HIV-1 challenges	[63]
	Cancer	IFNβ-NIS	tumor regression	[65,66]
		MUC1	tumor growth inhibition	[67]
**Measles virus**				
MV-Edm	ATC	MV-NIS	enhanced tumor killing	[68]
	ovarian CA	MV-SCD	lysis of primary tumors	[70]
	HCC	MV-SCD	apoptosis-like cell death	[70]
	CC	MV-SCD	prolonged survival	[71]
**NDV**				
NDV	melanoma	IL15	suppression of tumor growth	[72]
NDV90	lung CA	GFP	tumor-selective replication	[73]
NDV	HCC	IL2-TRAIL	prolonged survival	[74]
NDV Anhinga	HCC	TRAIL	tumor suppression	[75]
**Picornaviruses**				
Coxsackievirus				
CVB3	IN	FGF2	improved blood flow	[76]
CAV21	melanoma	ICAM1, DAF	tumor regression metastases	[77]
CAV21	breast CA	ICAM1, DAF	improved tumor regression	[78]
CAV21	breast CA	ICAM1,DAF+DH	tumor regression, dose dependence	[79]
CVA21, EV1	prostate CA	DAFv	superior tumor regression	[80]
**Poxviruses**				
PANVAC	pancreatic CA	MUC-1, CEA	induced CTL responses	[82]
VV	HCC	GLV-2b372	reduced tumor volume	[83]
VV-GLV-1h153	prostate CA	NIS + radiotherapy	restricted tumor growth, improved survival rate	[84]
VV	bladder CA	F4L-mutated	tumor regression, complete ablation	[85]
CPXV	glioblastoma	FCU1	tumor growth inhibition	[86]
CPXV	colon CA	FCU1	tumor growth inhibition	[86]

5-FC, 5-fluorocytisine; Ad, adenovirus; AAV, adeno-associated virus; AD, Alzheimer’s disease; ATC, anaplastic thyroid cancer; CA, carcinoma; CAV21, Coxsackievirus A21; CC, cholangiocarcinoma; CD, cytosine deaminase; CPVX, cowpox virus; CRISPR, clustered regularly interspaced short palindromic region; CT. cystic fibrosis; CTL, cytotoxic T lymphocyte; CVB3, Coxsackievirus B3; DAF, decay-accelerating factor; DH, doxorubicin hydrochloride; DMD, Duchenne muscular dystrophy; EV1, enteroviruses 1: FGF2, fibroblast growth factor-2; GRV, gammaretrovirus; HCC, hepatocellular carcinoma; HD, Huntington’s disease; HSV, herpes simplex virus; HTT, huntingtin; HIV, human immunodeficiency virus; ICAM-1, intercellular adhesion molecule-1; IL-12, interleukin-12; IL-24, interleukin-24: IN, ischemic necrosis; MDA-7, melanoma differentiation associated gene 7; MeCP2. Methyl CpG protein 2; miRNAs, micro-RNAs; MV-SCD, measles virus super-cytosine deaminase; NDV, Newcastle disease virus; PD, Parkinson’s disease; PDN, painful diabetic neuropathy; PKD, pyruvate kinase deficiency; PKLR, pyruvate kinase isoenzymes L/R; PNETs, pancreatic endocrine tumors; RRV, retroviral replicating vector; RTT, Rett Syndrome; SB, Sleeping Beauty; SHIV, Simian/Human Immunodeficiency Virus; SFV, Semliki Forest virus; SN, sensory neuropathy; VSV, vesicular stomatitis virus; X-CGD, X-linked chronic granulomatous disease; VV, vaccinia virus.

**Table 3:** Examples of clinical trials using viral vectors.

Disease	Viral Vector	Response	Reference
Hemophilia A	AAV-FVIII/FIX	Cure of hemophilia	[88,89]
Hemophilia B	Lenti-FVIII	Potential cure	[87]
	Lenti-FIX	Life-long production of FVIII	[90]
Cancer	Enadenotucirev	Good safety, no serious adverse events in phase I	[87]
	HSV HF10	Good safety, antitumor activity	[42]
	HSV HF10	Combination therapy anti-CTLA-4	[42]
HGG	Toca 511	Improved survival	[91]
	Toca 511/FC	Phase II/III trial in progress	[92]
Glioblastoma	HSV G207	Antitumor activity in Phase I	[97]
	HSV G207	Design of phase I trial for children with glioblastoma	[98]
CGD	Gamma RV	Resolution of infections, but malignant transformation	[48]
ATC	MV-NIS	Targeting iodine-resistant ATC	[66]
Colorectal CA	Oncolytic VV	Induction of immune response	[93]
	NDV	Prolonged survival of patients in phase II study	[107]
Kidney CA	LipoSFV-IL12	Transient IL-12, repeated injections	[102]
Pancreatic CA	PANVAC-VF	Failure in phase III, encouraging results in new phase I trial	[94,95]
Prostate CA	NDV-TAA	Improved survival in phase II	[104]
	VEE-PSMA	Neutralizing antibodies in phase I	[101]
Melanoma	NDV	Phase II/III failed to show superiority to control	[105]
	CVA21	Anti-tumor activity in melanoma patients	[111,112]
	CVA21 + PLMab	Overall response rate 60%, stable disease in 27% of patients	[113]
	LipoSFV-IL12	Transient IL-12, repeated injections	[102]
Solid tumors	NDV PV701	Progression-free survival	[106]
CF	Lenti-hCEF-CT	Expression, toxicity and integration profiles support for clinical trials	[114]

AAV, adeno-associated virus; ATC, anaplastic thyroid cancer; CF, cystic fibrosis; CGD, chronic granulomatous disease; CVA21, Coxsackievirus CVA21 strain; FIX, Factor IX; FVIII, Factor VIII; Gamma RV, gammaretrovirus; HGG, high-grade glioma; HIV, human immunodeficiency virus; HSV, herpes simplex virus; LipoSFV-IL12, liposome-encapsulated Semliki Forest virus-interleukin-12; MV-NIS, measles virus-sodium iodide symporter; NDV-TAA, Newcastle disease virus-tumor associated antigen; PANVAC-VF, vaccinia-fowlpox virus; PLMab, pembrolizumab; shRNA, short hairpin RNA; VEE, Venezuelan equine encephalitis virus; VV, vaccinia virus.
